# ORGANIZATIONAL AND COMMUNITY FACTORS ASSOCIATED WITH NURSING HOME ORGANIZATIONAL CULTURE

**DOI:** 10.1093/geroni/igz038.1883

**Published:** 2019-11-08

**Authors:** Akbar Ghiasi, Ganisher K Davlyatov, Justin C Lord, Robert Weech-Maldonado

**Affiliations:** 1 University of Alabama at Birmingham, Birmingham, Alabama, United States; 2 Louisiana State University at Shreveport, Shreveport, United States

## Abstract

This study examines how organizational and community factors are associated with organizational culture among high Medicaid nursing homes (70% or higher Medicaid census). According to the Competing Values, Framework, there are four types of organizational culture: clan culture (friendly working environment); adhocracy culture (dynamic/creative working environment); market culture (results-based organization); and hierarchy culture (formalized/structured work environment). Survey data from 324 nursing home administrators (30% response rate) from 2017- 2018 were merged with secondary data from LTCFocus, Area Health Resource File, and Medicare Cost Reports. The dependent variable consisted of organizational culture type. The independent variables comprised organizational factors (facility has nurse practitioner/physician assistant (NP/PA), RN/LPN/CNA hours per resident day, RN skill mix, ownership, chain affiliation, size, occupancy rate, and Medicare and Medicaid payer mix) and community factors (Medicare Advantage penetration, per capita income, educational level, unemployment rate, poverty level and competition). Multinomial regression results showed that, compared to facilities with hierarchical cultures, those with a market culture have greater odds of having an NP/PA and higher RN skill mix and LPN intensity, but lower odds for RN intensity, Medicaid payer mix, occupancy rate, and Black residents. Also compared to facilities with hierarchical cultures, those with a clan culture have lower odds for having an NP/PA, beds and occupancy rate, but higher odds of being located in communities with lower unemployment and higher Medicare managed care. In conclusion, different organizational cultures are associated with different staffing patterns, as well as organizational and community factors.

